# Relationship between Pressure and Output Parameters in Belt Grinding of Steels and Nickel Alloy

**DOI:** 10.3390/ma14164704

**Published:** 2021-08-20

**Authors:** Nelli Vladimirovna Syreyshchikova, Danil Yurievich Pimenov, Munish Kumar Gupta, Krzysztof Nadolny, Khaled Giasin, Muhammad Aamir, Shubham Sharma

**Affiliations:** 1 Department of Automated Mechanical Engineering, South Ural State University, Lenin Prosp. 76, 454080 Chelyabinsk, Russia; snv.ktn@mail.ru; 2 Faculty of Mechanical Engineering, Opole University of Technology, 45-758 Opole, Poland; munishguptanit@gmail.com; 3 Department of Production Engineering, Faculty of Mechanical Engineering, Koszalin University of Technology, Racławicka 15-17, 75-620 Koszalin, Poland; krzysztof.nadolny@tu.koszalin.pl; 4 School of Mechanical and Design Engineering, University of Portsmouth, Portsmouth PO1 3DJ, UK; Khaled.giasin@port.ac.uk; 5 School of Engineering, Edith Cowan University, Joondalup, WA 6027, Australia; m.aamir@ecu.edu.au; 6 Department of Mechanical Engineering, IK Gujral Punjab Technical University, Main Campus-Kapurthala, Punjab 144603, India; shubham543sharma@gmail.com

**Keywords:** belt grinding, machining, pressure, surface roughness, material removal rate (MRR), tool wear, steel, nickel alloy

## Abstract

Belt grinding of flat surfaces of typical parts made of steel and alloys, such as grooves, shoulders, ends, and long workpieces, is a good alternative to milling. Several factors can influence the belt grinding process of flat surfaces of metals, such as cutting speed and pressure. In this work, the importance of pressure in the belt grinding was investigated in terms of technological and experimental aspects. The grinding experiments were performed on structural alloy steel 30KhGSN2/30KhGSNA, structural carbon steel AISI 1045, corrosion-resistant and heat-resistant stainless steel AISI 321, and heat-resistant nickel alloy KHN77TYuR. The performance of the grinding belt was investigated in terms of surface roughness, material removal rate (MRR), grinding belt wear, performance index. Estimated indicators of the belt grinding process were developed: cutting ability; reduced cutting ability for belt grinding of steels and heat-resistant alloy. It was found that with an increase in pressure *p*, the surface roughness of the processed surface *Ra* decreased while the tool wear $V_{B}$ and MRR increased. With a decrease in plasticity and difficulty of machinability, the roughness, material removal rate, reduced cutting capacity (Performance index) $q_{per}$, material removal Q decreased, and the tool wear $V_{B}$ increased. The obtained research results can be used by technologists when creating belt grinding operations for steels and alloys to ensure the required performance is met.

## 1. Introduction

To satisfy the customer demands in terms of cost production and high-quality products, there is a huge need for developments in mechanical engineering, especially in the manufacturing sector [1,2]. In grinding operation, the product quality and abrasives used are highly important to achieve the desired production goals [3]. This is because belt grinding operations have a significant role at all stages of manufacturing [4,5]. Therefore, the widespread use of belt grinding operations has determined its high demand [6] in various industries, such as aerospace [7,8], machinery [9,10], automotive [11,12], rail grinding industry [13,14], etc. However, abrasive belts are often operated ineffectively due to the use of non-rational grinding modes [15]. The efficiency of flat grinding operation depends on many factors such as cutting mode [16], parameters of grinding operations (pressure) [17], workpiece material [18], etc. and the selection of these factors is essential from an industrial point of view. Previously, Wang et al. [19] proposed a belt grinding technique for complex surface machining, which accounted for pressure and showed the advantages of the non-linear material removal rate (MRR) model. Zhe et al. [20] studied the different contact pressures and their influence on the characteristics of belt rail grinding of U71Mn steel. They claimed that the increment in contact pressure had a positive effect on the MRR values and the hardness of the machine surface. Huang et al. [21] discussed the impact of various grinding parameters such as grinding pressure, liner speed, frequency, and time on the roughness of pump gear. Zhao et al. [22] studied the influence of belt rail grinding of AlSI 4340 steel on surface roughness and obtained a method for predicting roughness. Fan et al. [23] proposed a microscopic model of contact pressure to reveal the contact behavior of each active grain based on a digital representation of the surface topography with belt rail grinding of AlSI 4340 steel.

The above studies indicate that the majority of previous publications on belt grinding were devoted to machining, especially on the grinding of stainless steels [24,25] and alloys of increased strength and hardness [26], and to the main methods of profile grinding of parts such as blades, etc. [27,28,29]. The practical significance of belt grinding with stringent flat surface requirements is an alternative to milling. This is true for typical parts, such as grooves, ridges, ends, and long parts. However, flat belt grinding has been studied to a lesser degree. In our previous studies [15,30], the machinability and the influence of cutting speed during belt grinding of steels and alloys were investigated. However, the studies lack the effect of pressure for different groups of materials and alloys. Moreover, the standards for cutting modes with a grinding belt have not yet been developed. It is worth noting that future recommendations and the effect of individual parameters on machining operation are important. Therefore, in each specific case in production, manufacturers are forced to experimentally determine the suitability of the sanding belt and set the pressure by choosing the clamping force. These insignificant recommendations are not scientifically founded since the parametric effect of the belt grinding process with the belt properties on performance indicators has been rarely studied. Furthermore, the applied indicators recommended for the selection and purpose of grinding belts during operation are rarely discussed in the literature.

Research studies reported by S.N. Korchak [31], S. Malkin [32], P.P. Pereverzev [33], and others studied the influence of grinding parameters and characteristics of abrasive tools on the efficiency of the grinding process. This allowed for establishing the degree to which pressure influences processing results, thereby determining the increase in the adequacy of technological solutions when assigning grinding modes [34,35].

An analysis by Aurich and Kirsch [36], Yang et al. [37], and Dai et al. [38] on micro-cutting with single grains, grain morphology, the topography of the working surface of abrasive tools, and grain shape modeling, showed that it is possible to determine the main factors that must be considered when designing grinding operations with abrasive tools. These include the main grinding mode parameters (pressure and cutting speed) when assigning tool properties and achieving the necessary results determined by the established performance indicators [26,39]. In previous papers [15,30], the authors of this work investigated belt grinding of heat-resistant and stainless steels and alloys. The results showed that the effect of pressure on the output parameters of belt grinding of such materials has not been sufficiently reported in the open literature. It was also determined that there are significant contradictions in the choice of cutting modes, including belt grinding pressure.

Therefore, this work aims to improve the belt grinding of flat surfaces of metals and alloys using fabric grinding belts. The specific aim here is to enable manufacturers to use technical rationale when choosing belt pressure for surface grinding of steel and nickel alloy. To achieve this, the current study investigates and establishes dependences of efficiency indicators, such as surface roughness, material removal rate (MRR), grinding belt wear, and performance index, on the pressure during belt grinding of flat surfaces of steels and alloys.

## 2. Theoretical Provisions

An analysis of belt grinding pressures showed the data are insufficient and often contradictory. Reznikov et al. [40] considered pressures of 1.5∙10^5^–3.0∙10^5^ Pa to be suitable for finishing machining of carbon steel, and proposed 2.0∙10^5^–4.0∙10^5^ Pa for rough machining. Hamdi et al. [41] studied the influence of polymer contacting rollers and pressure on the surface texture finish in the belt grinding of 16MC5 casehardened steel. Gong et al. [42] showed the effect of grinding pressure and speed on productivity for belt grinding of titanium alloy. Abrasive belts made of new grinding materials are widely advertised: Cubitron [43], Cubicut [44], and Alundum [45,46] with wear-resistant bonds and on new polyester bases, which make belt grinding close to milling. However, there are no recommendations or studies on the choice of critical or permissible pressures on the working layer of abrasive belts for surface grinding of steels and heat-resistant alloy.

The methodology assessing the operational properties of the grinding belt was determined in a previous study [47]. It was decided to apply the indicators to the unit of the working surface of the belt (or contact between the belt and the workpiece). This made it possible to make the performance indicators comparable. In the development of performance indicators for the correct reflection of the physical essence of belt processing, a distinction was made between the selected indicators according to the purpose of the tool. The assessment of the operational properties of the tool for rough and finished grinding and polishing was taken differently due to different requirements for these operations. This was based on the position that the main purpose of roughing is to remove the stock in the optimal minimum time, while the goal of finishing and polishing is to achieve the required quality of the finished surface in the optimum minimum time. On this basis, it was decided to select indicators based on the operation being performed. The analysis showed that grinding pressure is the most important factor [47], with a dominant effect on the performance of the grinding belt processing process. The magnitude of the contact pressure depends on the pressing force and the contact area, which are influenced by the deformation of the abrasive tool and the contact roller and many other factors. We proposed a formula to determine the value of the contact pressure (*p*) of the tool and workpiece for flat belt grinding [47]:

(1) $$p=\frac{P_{y}\cdot 10^{5}}{L\cdot B\cdot K_{j}}\;,$$

where *P_y_* is the cutting force directed along the normal to the machinable surface (radial clamping force of the tool and workpiece); *L* is the contact length in the direction of belt movement; *B* is the contact width; and *Kj* is the coefficient depending on the deformation of the contact roller, material workpiece properties, the size of the stock, deformation of the belt and other factors. Comparable quantitative processing characteristics were selected, which reflect roughing and finishing operations as an interaction between contacting surfaces, for example, reduced cutting ability (performance index) ($q_{Per}$) [15,30,47]:

(2) $$q_{Per}=\frac{\sum_{1}^{n}q_{i}}{\tau \cdot P_{c}\cdot v_{c}},$$

where $q_{i}$ is the material removed over the i-th grinding period; $P_{c}$ is the clamping force of the tool to the workpiece; τ is the operating time of the tool until the resistance criterion (tool life); and $v_{c}$ is the cutting speed. $q_{Per}$ is the characteristic of stock removal from the workpiece per unit of work expended.

The surface roughness (Ra) was chosen as a comparable quantitative characteristic because it is applied in the evaluation of a large number of scratches to a workpiece surface [48,49,50,51].

It was assumed that the other test conditions were entirely and accurately reproduced: cutting speed, workpiece speed, characteristics of the workpiece being processed, the value of the initial roughness, and others, which ensured the comparability of the developed estimated indicators.

Tool quality and pressure—which are some of the key factors of the effectiveness and efficiency of the belt grinding process—were chosen as the objectives of the research. According to the revealed dependences of performance on the pressure during grinding, it is a requirement to apply grinding belts with certain properties for specific processing operations. Therefore, the requirements for the comparability of the estimated performance characteristics of the abrasive tool and the pressure test conditions were determined.

## 3. Materials and Methods

A cloth-backed sanding belt with grade 15A brown aluminum oxide was used in the current study, with a grain size F60, on a synthetic bond according to GOST 27181 (Russian Standard) and on a hide (natural) bond according to GOST 5009 (Russian Standard). The tests were carried out under controlled conditions with a change in the radial clamping force or the contact area of the tool and the workpiece; all other factors remained constant. The tests were carried out with clamping forces $P_{c}$ of 14.7–98.1, N. Other test conditions: cutting speed *v_c_* = 25 m/s; workpiece speed *v_w_* = 0.058 m/s; vertical oscillation frequency *w_os_* = 200 mm^−1^; the value of the vertical oscillation *Aos* = 3 mm. Table 1 shows the chemical composition and physical and mechanical properties of these steels and alloy for blanks [15,30].

**Figure 1:**
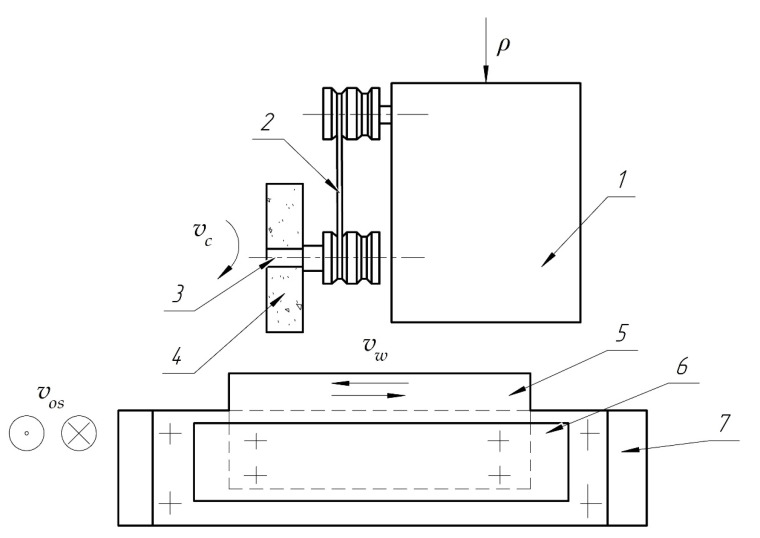
Belt grinding scheme: (1) machine head; (2) pulleys; (3) rollers mounting cartridge; (4) sanding belt; (5) blank; (6) support; (7) test bench frame; p pressure; vc belt speed; vw workpiece speed; wos vertical oscillation frequency.

The grinding belts were tested by simulating flat belt grinding with a contact roller (see Figure 1). An IS-78 model stand (ChOZ plant (Chelyabinsk Experimental Plant), Chelyabinsk Russia), created based on a modernized cylindrical grinding machine model 3110M (Tbilisi Grinding Machine Plant, Tbilisi, Georgia) was used for the tests. Additional details for the tests are provided in Table 2.

The surface roughness (R_a_) was measured using a surface roughness profilometer with a unified electronic AP system, model 263 (Proton JSC, Orel, Russia).

Tool wear $V_{B}$ (the mass of the worn-out working layer of the tape), is determined by the weight method on laboratory scales of the VLT type in accordance with GOST 19874 (Russian Standard) (LLC “Sartogosm”, St. Petersburg, Russia) with three repetitions; the dispersion is 0.02 and 0.01 and the coefficient of variation 17.7 and 6.7, respectively, with bluntness and destruction of the working layer.

The choice of the applied process parameters and their levels was determined by the prevailing practice of machining operations on belt grinding operations of flat surfaces. The applied process parameters were the clamping force of the tool and the workpiece, the cutting speed, the ratio of the densities of the processed metal and abrasive, the contact area of the tool, and blanks.

## 4. Results and Discussion

**Figure 2:**
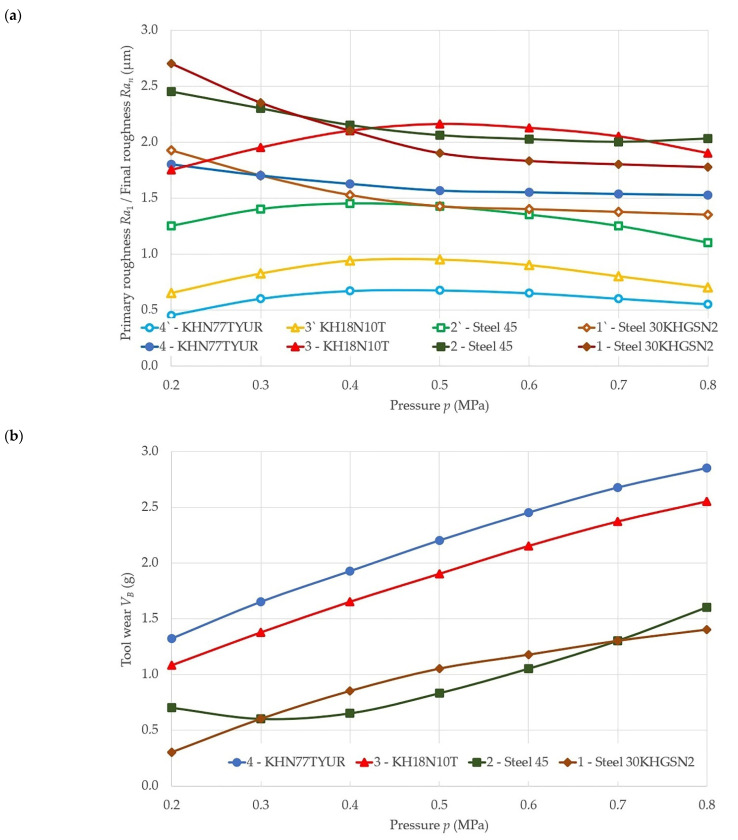

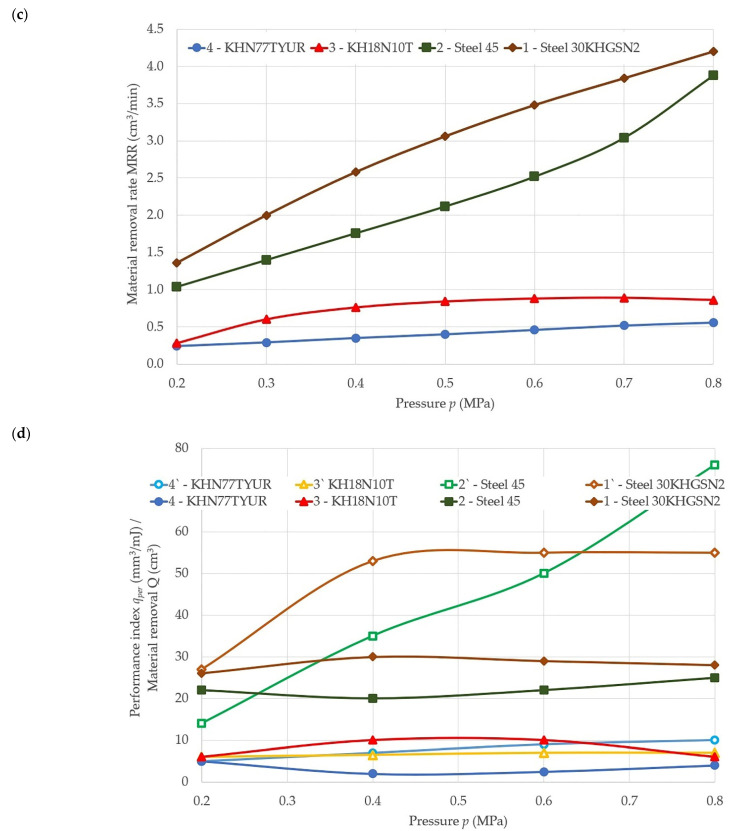
Dependences of the performance of the belt on the pressure when grinding steels and alloy of different grades: (**a**) $Ra_{1}$ the roughness of the processed surface after the first grinding cycle (primary roughness), μm; $Ra_{n}$ the roughness of the processed surface after the *n*-th grinding cycle (final roughness), μm; (**b**) $V_{B}$ tool wear, g; (**c**) Material removal rate (MRR), cm^3^/min; (**d**) Q material removal, cm^3^; and $q_{per}$ reduced cutting capacity, mm^3^/mJ: for the belt on a C synthetic bond: workpiece material steels 45, 30KhGSN2 and Kh18N10T and alloy KhN77TYuR; cutting speed *v_c_* = 25 m/s; workpiece speed *v_w_* = 0.058 m/s; vertical oscillation frequency *w_os_* = 200 mm^−^^1^; the value of the vertical oscillation *Aos* = 3 mm; grit = F60.

Figure 2 shows the dependencies of performance indicators ($Ra_{1}$ the roughness of the processed surface after the first grinding cycle (primary roughness), μm; $Ra_{n}$ the roughness of the processed surface after the *n*-th grinding cycle (final roughness), μm; $V_{B}$ tool wear, g; MRR Material removal rate, cm^3^/min; Q material removal, cm^3^; and $q_{per}$ reduced cutting capacity, mm^3^/mJ) of 14AF60C sanding belt from pressure (*p*, MPa) when grinding different metals: steels 30KHGSN2, 45, and Kh18N10T and alloy KhN77TYuR.

The analysis showed (see Figure 2a–c) that Q, MRR, and $V_{B}$ increase with increasing pressure. With a fourfold increase in pressure, *Q* and MRR increase more significantly (2.0–5.2 times) when processing 30KHGSN2 and 45 steel than when processing KH18N10T steel and KHN77TYuR alloy (1.1–3.0 times). Moreover, $V_{B}\;$is far less (0.73–1.5 g) when grinding 30KHGSN2 and 45 steel (steels with better machinability) than when grinding steels and alloys of the worst machinability—KH18N10T and KHN77TYUR (1.15–2.7 g). With an increase in pressure, belt wear increases slightly less when grinding easily machinable metals compared to difficult-to-machine ones: the curves of the wear dependence for 30KhGSM2 and 45 steels are flatter than for metals Kh18N10T and KhN77TYuR (see Figure 2b). With a fourfold increase in pressure, *Q* and MRR increase more significantly (2.0–5.2 times) when processing 30KHGSN2 and 45 steels than when processing grade KH18N10T steel and grade KHN77TYUR alloy (1.1–3.0 times). Moreover, the index of belt wear Δ when grinding steels of grades 30KhGSM2 and 45 (steels with better machinability) is far less (0.73–1.5 g) than when grinding steels and alloys of inferior machinability—Kh18N10T and KhN77TYuR (1.15–2.7 g). With an increase in pressure, the wear of the belt increases slightly less when grinding well-processed metals compared to difficult-to-machine ones: the curves of the wear dependence for steels of grades 30KhGSM2 and 45 are flatter than the curves of the dependence of wear for metals of grades Kh18N10T and KhN77TYuR (see Figure 2b).

Figure 2c,d shows the experimental dependences of *Q* and MRR on the pressure during belt grinding. With increasing pressure, the amount of material removed and the rate of removal increase. However, this growth does not always translate into improved tool productivity. The dependencies of the reduced cutting ability $q_{per}$ (see Figure 2) do not have the same direct proportionality observed for the dependencies of *Q*, MRR, and $V_{B}$. They do show certain pressure intervals at which an increase in metal removal per unit of time is achieved per unit of grinding energy expended. The $q_{per}\;$dependences show the different nature of the effect of pressure. Pressure is useful while metal removal increases due to the penetration of the cutting grains into the workpiece material, but pressure also creates conditions that destroy the contact between the bond and the grains of the sanding skin of the belt, destroying the tool grains. If the clamping force is so great that the resulting pressure causes creasing or dulling of the cutting edges of the grain or shelling, then pressure is playing a negative role and leads to a decrease in productivity and an increase in sanding belt wear. Rational pressure values for the studied metal grades were established by a combination of performance indicators: $q_{per}$, MRR, $V_{B}$, $Ra_{1}$, $Ra_{n}$.

In the investigated range of modes, it was established that the influence of the clamping force ($P_{C}$) and pressure (*p*) on the roughness of the machined surface after the first $Ra_{1}$ and last grinding cycle $Ra_{n}$ (see Figure 2a) is rather complex. In most cases, a decrease in Ra is observed with an increase in $P_{C}$, which is explained by an increase in the number of active grains and an increase in the contact area of the tool with the machined surface with an increase in ($P_{C}$). A decrease in $Ra_{1}$ and $Ra_{n}$ with increasing pressure is noted when using belts with a synthetic bond C and with a natural bond M (see Figure 2a). It was also found that $Ra_{1}$ and $Ra_{n}$, as a rule, decrease when moving from 30KHGSN2 and 45 steels to KH18N10T and KHN77TYUR steels and alloy, i.e., with an increase in the hardness of the metal, a decrease in ductility, and a deterioration in machinability (see Figure 2a).

**Figure 3:**
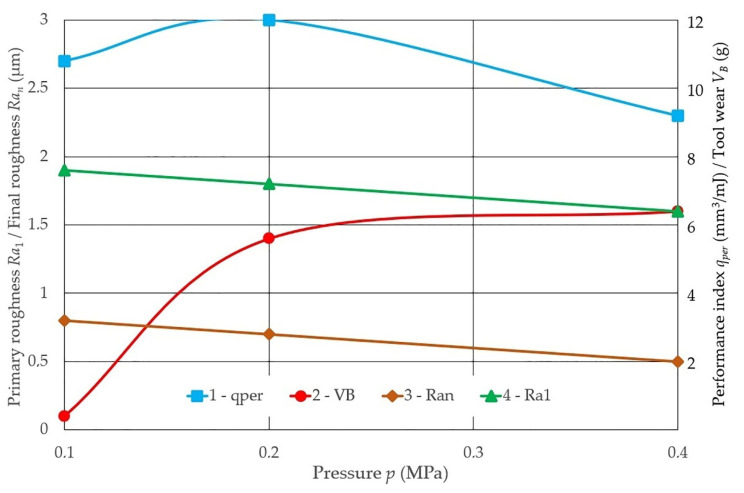
Dependences of the performance of the belt on the pressure when grinding Steel 45: 1 $q_{per}$; 2 $V_{B}$; 3 $Ra_{n}$; 4 $Ra_{1}$: workpiece material steel 45; for the belt on a C synthetic bond: workpiece material steel 45; cutting speed vc = 25 m/s; workpiece speed vw = 0.058 m/s; vertical oscillation frequency wos = 200 mm^−1^; the value of the vertical oscillation Aos = 3 mm; grit = F60.

Changes to the cutting ability $q_{per}$ differ in nature throughout operation τ with the 14AF60M belt at different grinding pressures of steel 45. This can be seen from the given dependences. For example, increasing the pressure from 0.1 to 0.2 MPa reduces the initial removal and shortens the first period of operation (running-in period), while increasing the pressure from 0.2 to 0.4 MPa sharply reduces belt durability (see Figure 3).

Equations (3)–(5) establish the functional dependences of the cutting capacity ($q_{per}$) of the grinding belt over the processing time (*τ*), described by the exponential for three values of the applied pressure values: *p* = 0.1 MPa, *p* = 0.2 MPa and *p* = 0.4 MPa:

(3) $$\mathrm{When}\;p\;=0.1мПa;\;q_{Per}=19,8\cdot e^{-0.05\cdot \tau }$$

(4) $$\mathrm{When}\;\;\;p\;=0.2мПa;\;q_{Per}=15,6\cdot e^{-0.03\cdot \tau };$$

(5) $$\mathrm{When}\;\;\;p\;=0.4мПa;\;q_{Per}=16,64\cdot e^{-0.16\cdot \tau }$$

The experimental range of pressure during grinding was conditionally divided into three zones according to the nature of the wear of the grinding belt (see Figure 3). The first range of pressures is characterized mainly by the bluntness of abrasive grains. The second range of pressures is characterized by the self-sharpening of grains and the shedding of the working layer of the belt. The third range of pressures during grinding is characterized by tearing of grains from the bond, destruction of the working layer of the belt down to the base and tool failure due to cutting of the base or belt breakage. The limits of the values of the grinding pressure for each of the ranges are different for different characteristics of the belt and depend on the substrate and the requirements for the quality of processing. Figure 3 illustrates three zones of belt wear from changes in pressure during grinding.

The established dependences of the performance indicators of the sanding belt show that under the same conditions (workpiece material, pressure, and other parameters of the grinding mode), belts on a synthetic bond are significantly more efficient: $q_{Per}$ is about 1.5 times higher, while $V_{B}$ is about nine times slower than the natural bond instrument shows.

## 5. Summary

Depending on the characteristics of the sanding belt, the pressure ranges during grinding can be determined by the nature of the tool wear. Tool wear increases with the increase in the grain size of the belt. Moreover, tool wear increases when changing from a natural to a synthetic bond (for a combined bond, the pressure values are intermediate values), and decrease with the transition from preliminary grinding to final grinding. The pressure ranges were also established considering the listed factors (characteristics of the grinding belt and the type of processing) for different groups of machinability of steels and alloys. It was found that increasing the pressures led to a complication in the machinability of the alloys.

The set sanding pressure range itself has three zones according to the wear pattern of the sanding belt with grit sizes F150 to F16. The first range of grinding pressures is characterized mainly by the bluntness of abrasive grains. The second range is characterized by the phenomena of self-sharpening of grains and shedding of the working layer of the belt. The third range of pressures during grinding is characterized by tearing of grains from the bond, destruction of the working layer of the belt down to the backing, and failure of the tool due to a cut through the base or belt breakage. The limits of the values of the grinding pressure for each of the ranges differ based on the characteristics of the belt and depend on the material and the requirements for processing quality.

An analytical model was developed to determine the contact pressure between a tool and a workpiece for flat belt grinding. The model takes into account the contact between the tool and the workpiece, deformations of the contact roller, properties of the processed material, stock size, deformation of the grinding belt, and other factors.

The empirical dependence of the operating indicators (cutting capacity of the belt) on the grinding time was calculated. The calculations, selection, and application of performance indicators for assessing the belt grinding process have been determined. In addition, collected statistics of performance indicators for the dependences of pressure during grinding on the characteristics of the grinding belt, on the type of processing, and the substrate were reported. The experiments allowed the determination of the empirical dependences of pressure during belt grinding for groups of machinability of steels and alloys with a grinding belt on the characteristics of the grinding belt and the type of processing. These dependencies were assessed. The error of approximation of the established dependences is no more than 3.3%–5.7%. The pressure value for belt grinding is set to tool wear, which is described by exponential dependencies. Adequate sensitivity and distinguishability of estimates has been achieved. The stability of the results obtained is at the required level and does not exceed 5%–6%.

It was evident that the belt performance is influenced by the grinding pressure. Recommendations were given for choosing the appropriate pressure (*p*, Pa) depending on the type of processing (preliminary or final grinding), on the group of metal machinability (from easy-to-machine to hard-to-machine), and the main parameters of the characteristics of the sanding belt (grain size, base, and type of bond).

## 6. Conclusions

The current study provides developed and experimentally established dependencies on the belt grinding factor. This makes it possible to use scientific justification when choosing and assigning the main technological parameter of the belt grinding process—i.e., pressure.

It was found that increasing the pressure *p* decreased the surface roughness *Ra*, increased the tool wear $V_{B}$ and MRR. This was mainly due to the increase in the number of active grains and an increase in the contact area of the tool with the machined surface. With a decrease in plasticity and an increase in the difficulty of machinability, the roughness, material removal rate, reduced cutting capacity (Performance index) $q_{per}$, material removal Q decrease, and the tool wear $V_{B}$ increases. The functional dependences of the cutting ability of the grinding belt in the processing time, described by the exponential for certain values of the applied pressures, were obtained.

For belt grinding, the empirical dependence of the operating parameters (cutting capacity of the belt) is calculated. Based on the established empirical dependencies of pressure, we developed recommendations for the selection and application of belt grinding pressure in the ranges covering the processes of surface grinding and for the machinability groups of steels and alloys. The recommendation here is to consider the chosen material workpiece, the main characteristics of the tool, and the operating parameters of the equipment used. The recommendations are of significant practical importance and can be applied by abrasives enterprises and consumer enterprises.

## Figures and Tables

**Table 1:** The chemical compositions and physical and mechanical properties of materials [15,30].

Material Group.	Workpiece Material	Physical and Mechanical Properties				Physical and Mechanical Properties														
		Yield Stress, *σ_y_,* MPa	Ultimate Stress, *σ_D_*, MPa	Density, *ρ*, kg/m^3^	Hardness, HB	Carbon, C	Silicon, Si	Manganese, Mn	Nickel, Ni	Sulfur, S	Phosphorus, P	Chromium, Cr	Cerium, Ce	Titanium, Ti	Boron, B	Lead, Pb	Iron, Fe	Aluminum, Al	Copper, Cu	Arsenic, As
Structural alloy steel	30KHGSN2 (30KHGSNA)	1375	1620	7770	255	0.27–0.34	0.9–1.2	1–1.3	1.4–1.8	to 0.025	to 0.025	0.9–1.2	-	-	-	-	≈95	-	to 0.3	-
Structural carbon steel	45 (analog of AISI 1045)	355	600	7826	207	0.42–0.5	0.17–0.37	0.5–0.8	to 0.25	to 0.04	to 0.035	to 0.25	-	-	-	-	≈97	-	to 0.25	to 0.08
Corrosion- and heat-resistant stainless steel	KH18N10T (analog of AISI 321)	196	510	7920	179	to 0.12	to 0.8	to 2.0	9–11	to 0.02	to 0.035	17–19	-	0.6–0.8	-	-	≈68	-	-	-
Heat-resistant nickel alloy	KHN77TYuR	650	1000	8200	255–321	to 0.07	to 0.6	to 0.4	70.076–77.4	to 0.007	to 0.015	19–22	to 0.02	2.4–2.8	to 0.01	to 0.001	to 1	0.6–1	-	-

**Table 2:** Belt grinding parameters for the experiment [15,30].

Belt speed $v_{c}$ (m/s)	25
Pressure p (MPa)	0.2, 0.4, and 0.8
Machined materials	Steels 45, 30KhGSN2, Kh18N10T, KhN77TYuR alloy

## Data Availability

All data generated or analyzed during this study are included in this article.

## Nomenclature

*A_os_*	Amplitude of the vertical oscillation
*Ra*	Arithmetic mean deviation of the assessed profile (surface roughness)
_*Vb*_	Belt speed
*P_c_*	Clamping force
*v_c_*	Cutting speed
*ρ*	Density
$V_{B}$	Grain blunting area (flank wear)
HB	Hardness
$v_{s}$	Longitudinal feed rate
*Q*	Material removal
MRR	Material removal rate
$q_{i}$	Material removal rate over the i-th grinding period
τ	Operating time of the tool till the resistance criterion (tool life)
$q_{Per}$	Performance index
*p*	Pressure
*P_y_*	Radial cutting force
$Ra_{1}$	Surface roughness after the first grinding cycle
$Ra_{n}$	Surface roughness after the n-th grinding cycle
*σ_D_*	Ultimate stress
*w_os_*	Vertical oscillation frequency
_Vw_	Workpiece speed
*σ_y_*	Yield stress
